# Freeze-drying: an alternative method for the analysis of volatile organic compounds in the headspace of urine samples using solid phase micro-extraction coupled to gas chromatography - mass spectrometry

**DOI:** 10.1186/s13065-016-0155-2

**Published:** 2016-03-01

**Authors:** Raphael B. M. Aggio, Arno Mayor, Séamus Coyle, Sophie Reade, Tanzeela Khalid, Norman M. Ratcliffe, Chris S. J. Probert

**Affiliations:** Department of Cellular and Molecular Physiology, Institute of Translational Medicine, University of Liverpool, Crown Street, L693BX Liverpool, UK; Marie Curie Palliative Care Institute Liverpool, University of Liverpool, London Road, L39TA Liverpool, UK; Faculty of Health and Applied Sciences, Frenchay Campus, University of the West of England, Coldharbour Lane, BS161QY Bristol, UK; Department of Surgery and Cancer, South Kensington Campus, Imperial College London, SW72AZ London, UK

**Keywords:** Metabolomics, VOC, SPME, GC-MS, Volatile organic compounds, Urine, Freeze-dry

## Abstract

**Background:**

Volatile organic compounds (VOCs) can be intermediates of metabolic pathways and their levels in biological samples may provide a better understanding about diseases in addition to potential methods for diagnosis. Headspace analysis of VOCs in urine samples using solid phase micro extraction (SPME) coupled to gas chromatography - mass spectrometry (GC-MS) is one of the most used techniques. However, it generally produces a limited profile of VOCs if applied to fresh urine. Sample preparation methods, such as addition of salt, base or acid, have been developed to improve the headspace-SPME-GC-MS analysis of VOCs in urine samples. These methods result in a richer profile of VOCs, however, they may also add potential contaminants to the urine samples, result in increased variability introduced by manually processing the samples and promote degradation of metabolites due to extreme pH levels. Here, we evaluated if freeze-drying can be considered an alternative sample preparation method for headspace-SPME-GC-MS analysis of urine samples.

**Results:**

We collected urine from three volunteers and compared the performances of freeze-drying, addition of acid (HCl), addition of base (NaOH), addition of salt (NaCl), fresh urine and frozen urine when identifying and quantifying metabolites in 4 ml samples. Freeze-drying and addition of acid produced a significantly higher number of VOCs identified than any other method, with freeze-drying covering a slightly higher number of chemical classes, showing an improved repeatability and reducing siloxane impurities.

**Conclusion:**

In this work we compared the performance of sample preparation methods for the SPME-GC-MS analysis of urine samples. To the best of our knowledge, this is the first study evaluating the potential of freeze-dry as an alternative sample preparation method. Our results indicate that freeze-drying has potential to be used as an alternative method for the SPME-GC-MS analysis of urine samples. Additional studies using internal standard, synthetic urine and calibration curves will allow a more precise quantification of metabolites and additional comparisons between methods.

**Graphical abstract:**
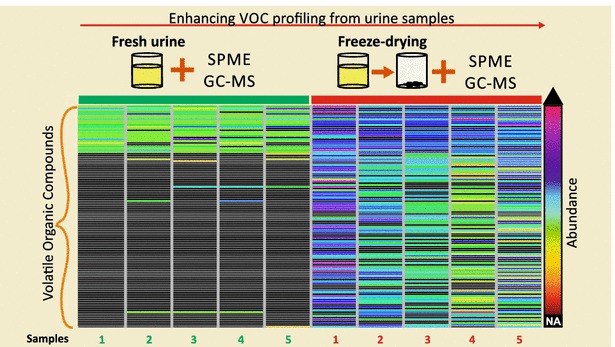
Enhancing VOC profiling from urine samples.

**Electronic supplementary material:**

The online version of this article (doi10.1186/s13065-016-0155-2) contains supplementary material, which is avaialble to authorize users.

## Background

Volatile organic compounds (VOCs) represent a chemically diverse group of metabolites found in biological fluids, with a boiling point lower than 300 °C and generally containing less than 12 carbon atoms [1]. VOCs are intermediates of metabolic pathways and, thus, their concentrations are likely to change when the metabolism of a cell or an organism reaches a different metabolic state [2]. Therefore, the levels of VOCs in biological samples may provide a better understanding of mechanisms driving cellular processes, diagnose diseases and/or monitor their progression [3].

A diverse range of analytical methods, such as electronic noses [4], selected ion flow tube mass spectrometry [5] and gas chromatography - mass spectrometry (GC-MS) [6], have been used to analyse VOCs in urine samples. Among them, GC-MS is perhaps one of the most popular [6]. Coupled to solid-phase micro extraction (SPME), it is possible to detect VOCs present in the headspace of urine samples [7]. The SPME fibre extracts metabolites, while the GC-MS performs both their separation and detection.

The headspace-SPME-GC-MS analysis of fresh urine samples generally produces a limited profile of VOCs. Thus, several sample preparation methods have been proposed to enhance VOC profiling [6, 8]. The addition of salt (e.g NaCl), acid (e.g. HCl) or base (e.g. NaOH) solutions are the most common [9, 10]. In general, these sample preparation methods are expected to increase the concentration of compounds in the headspace of the urine samples by increasing the ionic strength of these samples, which result in a richer profile of VOCs detected by GC-MS [11].

Although the addition of salt, acid or base have been largely applied to the analysis of urine samples using headspace-SPME-GC-MS [12], they have some disadvantages that may be critical according to the type of study being performed. First, there is not yet a well-established method or protocol for analysing VOCs in urine using headspace-SPME-GC-MS. Different laboratories use different urine sample volumes and particular volumes and concentrations of salt, acid or base solutions [13], which, ultimately, restricts the comparison of results across studies. Second, the salt, acid or base solutions added to the urine samples might contain impurities, which represent an extra source of variability potentially misleading the final biological interpretation. Third, extremes of pH coupled to the temperature used in the SPME extraction (e.g. 60 °C) may promote further reactions involving compounds in the urine [6]. These reactions potentially produce secondary volatile and non-volatile compounds. In this case, the VOC profiles reported by GC-MS will not represent the metabolite content of the urine sample at its sampling time. Finally, the GC-MS analysis of solutions at extreme pH levels may promote the degradation of the GC column, which, consequently, shortens its lifetime and reduces the reproducibility across replicates (http://www.chromacademy.com/troubleshooter-gc/resources/gc-phenomenex-troubleshooting-1). Extreme pH may lead to SPME fibre and septum degradation. Water can also promote degradation. Therefore, there is a need for an improved sample preparation method that produces a reliable VOC profile of urine samples analysed by headspace-SPME-GC-MS without degrading the column.

Freeze-drying is a dehydration process widely used in biochemistry studies [14], metabolomics studies [15] and in the industry for preserving perishable material [16]. In summary, the freeze-drying process is able to remove water from the material being processed while keeping it frozen. For metabolomics studies, it represents the ability of dehydrating samples without degrading metabolites. Here, we evaluate if freeze-drying can be considered an alternative sample preparation method for the analysis of urine samples using headspace-SPME-GC-MS. For this, we compared a number of sample preparation methods. The number of VOCs identified, their chemical classes and the repeatability of their quantification were assessed when 4 ml urine samples from healthy volunteers were analysed fresh, frozen at −80 °C, freeze-dried, with the addition of 1 ml of saturated NaCl solution (salt), with the addition of 1 ml of 5M HCl solution (acid) or with the addition of 1 ml of 5M NaOH solution (base). The results obtained here indicate that freeze-drying may be considered an alternative sample preparation method for SPME-GC-MS analysis of urine samples.

## Results and discussion

### Stability of headspace-SPME-GC-MS

**Fig. 1:**
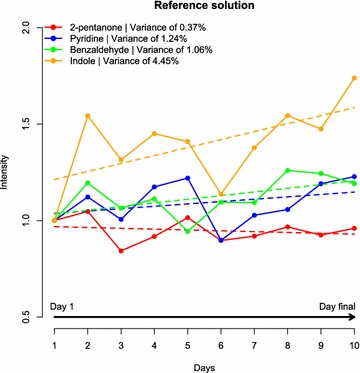
Intensity of compounds present in the reference solution throughout the experiment. A stock of reference solution was prepared at the beginning of the experiment. A 2 ml sample of the reference solution was analysed on the same days that urine samples were processed. The intensities of reference compounds were normalized by their intensities detected on day 1

The stability of the headspace-SPME-GC-MS system used in this study was assessed with the use of a reference solution containing four compounds (Fig. 1). Most compounds showed a variance in intensity of less than 1.3. Indole, however, showed a variance of 4.45. The explanation for this variation is that indole is a compound detected at a high retention time (i.e. 39.57 min), which is a region of the chromatogram that generally shows a higher level of variation due to column bleed [17]. In addition, the stock solution of standards was kept at room temperature, which may have resulted in oxidation of indole. The urine samples were all randomly analysed by headspace-SPME-GC-MS. Therefore, any compound showing the same variation as indole along the time frame of this experiment would equally affect every sample preparation method tested.

### Compound identification

**Fig. 2:**
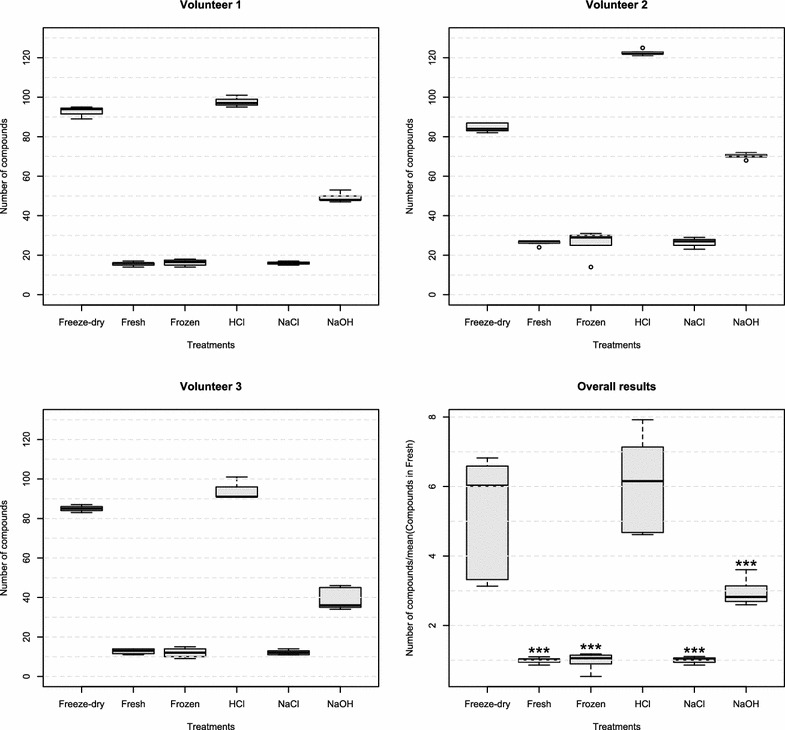
Number of compounds identified per treatment for each volunteer. For the overall results, Mann–Whitney U test was applied to compare the number of compounds identified by Freeze-dry in relation to all the other treatments tested. (*) p value < 0.05; (**) p value < 0.01; (***) p value < 0.001; n ≥ 3

**Fig. 3:**
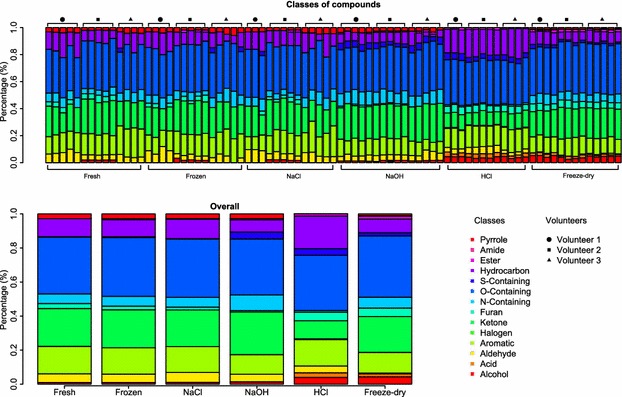
Chemical classes of compounds per treatment

In order to assess the performance of each treatment in recovering or extracting metabolites from urine samples, we compared the number of compounds identified across treatments and assessed the classes of compounds more likely to be extracted per treatment. Table 1 summarises the ratio of compounds identified per treatment in relation to compounds identified in fresh samples. Figure 2 demonstrates that Freeze-dry and the addition of HCl produced a significantly higher number of VOCs identified than any other treatment (Freeze-dry vs Fresh, p < 0.001; Freeze-dry vs HCl, p = 0.231; Freeze-dry vs NaCl, p < 0.001; Freeze-dry vs NaOH, p < 0.001; Freeze-dry vs Frozen, p < 0.001; HCl vs Fresh, p < 0.001; HCl vs NaCl, p < 0.001; HCl vs NaOH, p < 0.001; HCl vs Frozen, p < 0.001; Mann–Whitney U test), while there is no significant difference between Freeze-dry and HCl. For some samples, Freeze-dry and HCl reported six times more VOCs than Fresh, Frozen or NaCl, and 2–3 times more VOCs than NaOH. Figure 3 and Table 2 show a summary of the classes of compounds extracted by each treatment tested. Between compounds identified, Freeze-dry, HCl and NaOH recovered the most diverse classes of compounds. Compared to HCl, Freeze-dry detected a lower average number of acids, aldehydes, aromatics, halogens, furans, sulfur containing compounds, and hydrocarbons per sample; while it detected a higher average number of ketones, amides, pyrroles and other nitrogen containing compounds per sample. Compared to NaOH, Freeze-dry detected an equal or higher average number of compounds belonging to most classes per sample, with the exception of aldehydes and pyrroles. The differences in the classes of compounds recovered between each treatment tested (Table 2) are probably due to multiple factors. We know that the migration of compounds from the urine, or liquid phase, to the headspace of the vial, or gas phase, depends on the volume of the liquid phase, the volume of the gas phase and the affinity of compounds for the liquid and gas phases [18]. For example, the addition of salt is known to modify the matrix of the sample by increasing ionic activity. It decreases the solubility of compounds in the liquid phase, which results in more compounds moving to the gas phase. It is known that the addition of acid reduces the pH of the solution and increases the volatility of acids, while the addition of base increases the pH of the solution and increases the volatility of bases [18]. To the best of our knowledge, there is no work in the literature suggesting or discussing about headspace-SPME-GC-MS analysis of freeze-dried urine. Based on the results we found, we hypothesize that Freeze-dry improves the recovery of VOCs in urine samples by changing the volumes of the liquid and the gas phases. Freeze-drying considerably reduces the volume of the liquid phase while it increases the volume of the gas phase, which seems to promote the migration of compounds to the headspace of the vial. This mirrors the fact that small difference were observed in VOCs recovered by freeze-drying and other drying methods, such as air drying, high temperature drying (80 °C to 120 °C) and vacuum drying, when treating samples of ginger [19] and Wuyi Rock tea [20]. Furthermore, some of the compounds detected when using HCl or NaOH may result from compound degradation potentially promoted by the extreme pH levels of these solutions coupled to the high temperatures involved in the SPME-GC-MS analysis. In addition, although Freeze-dry was able to recover a higher number of compounds from a wider range of chemical classes, VOCs may be lost during the freeze-drying process. Additional experiments will allow us to further understand the chemistry and physics behind the headspace-SPME-GC-MS analysis of freeze-dried urine samples.

**Table 1:** Ratio of compounds identified per treatment tested in relation to fresh samples

Treatment	Mean	Median	S.E.	Mann–Whitney
Freeze-dry	5.18	6.03	0.45	–
Fresh	1.00	1.03	0.02	<0.001
HCl	5.96	6.15	0.35	0.231
NaCl	0.99	1.02	0.02	<0.001
NaOH	2.97	2.82	0.09	<0.001
Frozen	0.99	1.06	0.05	<0.001

The number of compounds identified by each treatment were normalized by the average number of compounds identified in urine samples analysed fresh (SE = standard error). Mann–Whitney U test compared the number of metabolites reported by each treatment in relation to Freeze-dry

### Compound quantification

**Fig. 4:**
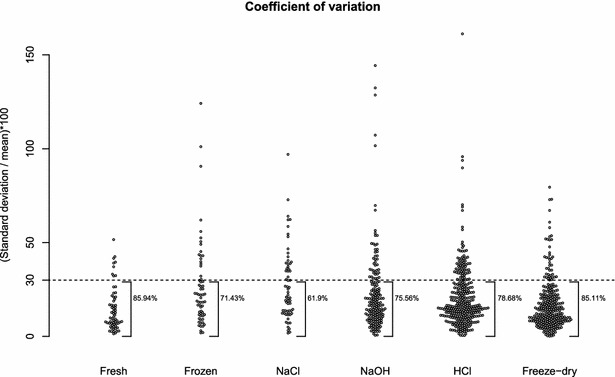
Coefficient of variation of metabolites identified when testing the different sample preparation methods. The coefficient of variations were calculated per volunteer before plotting. Each *dot* represents a single metabolite

In metabolomics, a coefficient of variation (CV) of 30 % is generally accepted as a variation threshold [21]. It is not recommended to draw biological interpretations based on compounds showing a CV higher than 30 %. Figure 4 shows the distribution of the CVs calculated for the compounds identified by each treatment tested in this study. Freeze-dry and Fresh were the most reproducible treatments (Fig. 4), with 85.11 and 85.94 % of metabolites associated with CVs lower than 30 %, respectively. Surprisingly, Frozen (71.43 %) showed significantly less repeatability when compared to Fresh (85.94 %) (p = 0.043; prop.test). It may be a result of the extra steps performed on Frozen samples (e.g. freezing and defrosting). NaCl, NaOH and HCl showed 61.9 %, 75.6 % and 78.7 % of compounds with CV lower than 30 %, respectively. The statistical comparisons of compounds with CV lower than 30 % across treatments is presented in Table 3. A single or multiple internal standards are generally applied for reducing the variability that may be introduced in the system during sample preparation. The best compound, or compounds, to be used as internal standard may vary according to the method used, the type of sample being analysed and the classes of metabolites being targeted. To the best of our knowledge, this is the first metabolomics study testing Freeze-dry as a sample preparation method for the headspace-SPME-GC-MS analysis of urine samples. In this study we generated the first SPME-GC-MS profile of metabolites found in freeze-dried human urine. Thus, no internal standard could be applied in this study. We are currently designing additional experiments to identify the best metabolites to be used as internal standard when freeze-drying urine samples.

**Table 2:** Average number of compounds identified per sample per class of compound and treatment tested (n ≥ 12)

Class	Fresh	Frozen	NaCl	NaOH	HCl	Freeze-dry	Mann–Whitney | p values				
							A vs. F	B vs. F	C vs. F	D vs. F	E vs. F
Alcohol	1	1	1	1.2	7.7	7.5	0.001	0.001	0.001	<0.001	0.889
Acid	0	0	0	1	5.3	3.1	NA	NA	NA	0.112	0.001
Aldehyde	1.9	1.9	2.2	4	7.8	1.1	0.002	0.009	0.003	<0.001	<0.001
Aromatic	5.9	5.6	5.5	10.3	30.2	21.3	<0.001	<0.001	<0.001	<0.001	<0.001
Halogen	0	0	0	0	1	0	NA	NA	NA	NA	NA
Ketone	8.1	8.1	7.9	22.6	20.6	36.9	<0.001	<0.001	<0.001	<0.001	<0.001
Furan	1.7	1.6	1.3	1.1	9.8	8.7	<0.001	<0.001	0.001	<0.001	0.048
N-Containing*	2.1	2.1	2.1	8.3	2	11.2	<0.001	<0.001	<0.001	0.003	<0.001
O-Containing**	12.2	12.5	12.5	29.6	62.9	63.2	<0.001	<0.001	<0.001	<0.001	0.849
S-Containing***	1	1	1	3.6	7.7	3.2	0.124	0.029	0.124	0.203	<0.001
Hydrocarbon	3.9	3.7	4.2	6.5	37.2	14	<0.001	<0.001	<0.001	<0.001	<0.001
Ester	0	1	1	1	2.3	2.9	NA	0.007	0.024	<0.001	0.198
Amide	0	0	0	0	0	1	NA	NA	NA	NA	NA
Pyrrole	1	1	1	2.5	0	1.8	<0.001	<0.001	<0.001	0.011	NA

*A* Fresh; *B* Frozen; *C* NaCl; *D* NaOH; *E* HCl; *F* Freeze-dry

* *N-Containing* Nitrogen containing compounds

** *O-Containing* Oxygen containing compounds

*** *S-Containing* Sulfur containing compounds

### Sample effects on chromatography

**Fig. 5:**
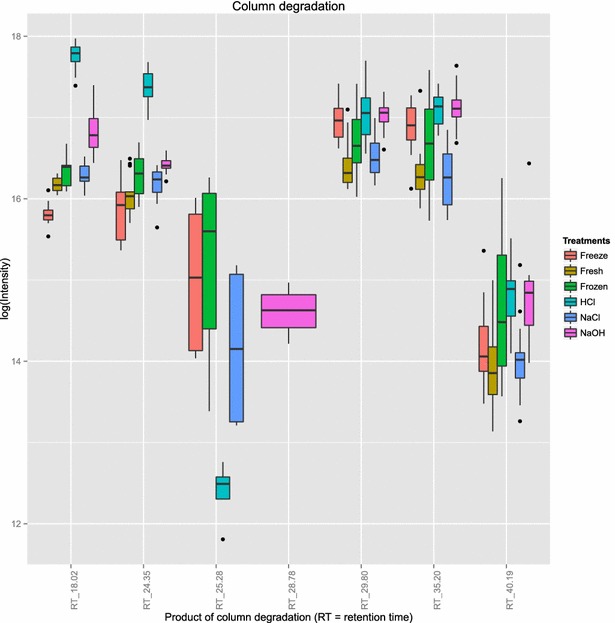
Gas chromatography column degradation. This figure shows the relative abundances of identified siloxanes per treatment tested. Compound IDs are based on their retention times

The type of samples or treatment applied to a sample prior to headspace-SPME-GC-MS analysis may result in a higher or lower degradation of the SPME fibre, septum, or GC column *(http://www.chromacademy.com/troubleshooter-gc/resources/gc-phenomenex-troubleshooting-1)*. Figure 5 shows the abundance of compounds originating from apparent polysiloxane degradation according to the treatment applied. Polydimethylsiloxane, for instance, is used in septa, SPME fibre and stationary phase used here. Table 4 shows their identifications and the results of statistical comparisons. Compared to HCl and NaOH, Freeze-dry resulted in detection of significantly lower or equal level of all column degradation products, with the exception of Phenyl-pentamethyl-disiloxane (RT_25.28). Compared to Fresh and NaCl, Freeze-dry showed higher abundances of two compounds, lower abundance of one compound and the same abundances of four compounds (Fig. 5). These results indicate that Freeze-dry does not promote further column degradation than any other method tested.

**Table 3:** Comparison of compounds showing coefficient of variation lower than 30 % and Freeze-dry

	Prop.test		Prop.test
Comparison	p value	Comparison	p value
Freeze vs Fresh	0.768	HCl vs NaOH	0.489
Freeze vs Frozen	0.016	NaOH vs Fresh	0.068
Freeze vs NaCl	<0.001	NaOH vs Frozen	0.632
Freeze vs NaOH	0.014	NaOH vs NaCl	0.056
Freeze vs HCl	0.052	NaCl vs Fresh	0.002
HCl vs Fresh	0.148	NaCl vs Frozen	0.345
HCl vs Frozen	0.272	Frozen vs Fresh	0.043
HCl vs NaCl	0.007		

### Practicalities in sample processing

Once HCl and NaOH solutions are prepared and ready to use, they can be quickly added to urine samples prior to headspace-SPME-GC-MS analysis. However, it requires someone manually adding these solutions to urine samples and extra care has to be taken to avoid contamination of these chemical solutions throughout the experiment, which would introduce variability to the system and potentially mislead the biological interpretation. The SPME-GC-MS analysis of the pure compounds (i.e. salt, base or acid) can certainly be performed to potentially identify contaminants, however, it results in additional time and cost associated to the study being performed. On the other hand, Freeze-dry is safe and only requires a freeze-drier machine. No specialized skills, other than operating a freeze-drier machine, are necessary and there is a very low risk of sample contamination. In this study, we freeze-dried samples for 18 h in order to assure that samples would be completely dry. However, this freeze-drying time can certainly be reduced depending on the freeze-drying machine available. In addition, Freeze-dry is considerably less labour intensive than NaOH, HCl or NaCl, and researchers are free to perform any other task while samples are freeze-drying. Furthermore, the experiment presented here was performed using the same GC-MS configuration for every treatment tested, which included a delay of 4 min prior to MS detection. This delay is generally applied to avoid the water peak. Freeze-dried samples contain no water, therefore, this delay may be removed, which potentially results in additional compounds being detected. Further experiments will allow to confirm or reject this hypothesis. Additionally, although tested in urine, the method suggested here could be applied to other complex aqueous samples such as waste water and food samples.

**Table 4:** Statistics of column degradation (n $\ge$ 12). IDs are based on the retention time of each metabolite

IDs	Name	CAS	ANOVA	tTest | p values				
				A–B	A–C	A–D	A–E	A–F
RT_18.02	Cyclotrisiloxane, hexamethyl-	541-05-9	0.000	0.000	0.000	0.000	0.000	0.000
RT_24.35	Cyclotetrasiloxane, octamethyl-	556-67-2	0.000	0.091	0.000	0.010	0.000	0.001
RT_25.28	Phenyl-pentamethyl-disiloxane	14920-92-4	0.001	–	0.000	0.240	–	0.775
RT_28.78	Diisopropyl(ethoxy)silane	90633-16-2	–	NA	NA	NA	–	NA
RT_29.80	Cyclopentasiloxane, decamethyl-	541-02-6	0.000	0.000	0.591	0.000	0.370	0.095
RT_35.20	Cyclohexasiloxane, dodecamethyl-	540-97-6	0.000	0.000	0.031	0.000	0.032	0.188
RT_40.19	Isopropoxy*	71579-69-6	0.000	0.246	0.005	0.449	0.013	0.115

*A* Freeze-dry; *B* Fresh; *C* HCl; *D* NaCl; *E* NaOH; *F* Frozen

*-* Identified in a single treatment

*NA* not identified in any of the tested treatments

* Isopropoxy-1,1,1,7,7,7-hexamethyl-3,5,5-tris(trimethylsiloxy)tetrasiloxane

## Experimental

### Stock solutions

Sodium chloride (NaCL, ≥99.5 %) and hydrochloric acid (HCl, >37 %) were obtained from Sigma Aldrich, Dorset, UK. Sodium hydroxide (NaOH, >97 %) was obtained from BDH limited, Poole, UK. A 100 ml stock solution of saturated sodium chloride (salt solution) was prepared with distilled water and 38 g of NaCl; a 100 ml stock solution of 5M sodium hydroxide (base solution) was prepared with distilled water and 20 g of NaOH; and a 100 ml stock solution of 5M hydrochloric acid (acid solution) was prepared by diluting concentrated hydrochloric acid with distilled water.

### Volunteer recruitment

The study presented here was performed in accordance with the Declaration of Helsinki and ethical approval was obtained from the North Wales Research Ethics Committee—West (REC reference number 13/WA/0266) with the Royal Liverpool and Broadgreen University Hospitals as research sponsor. Three healthy volunteers were recruited after obtaining informed consent. Volunteers were healthy male subjects of age 29 ± 2.5 years old (mean ± standard deviation) who were taking no medication for at least 4 months prior to sample collection.

### Urine samples

A 200 ml sample of first pass urine was collected from each volunteer and quickly divided into 30 aliquots of 4 ml stored in 10 ml vials for SPME-GC-MS analysis (Sigma-Aldrich, Dorset, UK). From these, twenty-five aliquots were stored at −80 °C while five aliquots were kept at room temperature to be analysed within 15 h of sample collection.

### Freeze-drying

An Edwards EF4 Modulyo freeze-dryer (Edwards High Vacuum, UK) operated at −35 °C and eight mbar was used to freeze-dry for 18 h five aliquots of 4 ml urine samples from each volunteer previously frozen at −80 °C.

### Headspace-SPME-GC-MS analysis

A Perkin Elmer Clarus 500 GC/MS quadruple bench top system (Beaconsfield, UK) was used in combination with a Combi PAL auto-sampler (CTC Analytics, Switzerland) for the analysis of all samples. The GC column used was a Zebron ZB-624 with inner diameter 0.25 mm, length 60 m, film thickness 1.4 $\mu$m (Phenomenex, Maccles field, UK). The carrier gas used was helium of 99.996 % purity (BOC, Sheffield, UK). A CAR-PDMS 85 $\mu$m fibre was used to extract VOCs from the headspace air above the samples for 20 minutes (Sigma-Aldrich, Dorset, UK). The fibre was pre-conditioned before use, in accordance with the manufacturer manual. Urine samples were placed in an incubation chamber at 60 °C for 30 min before fibre adsorption. The fibre desorption conditions were 5 min at 220 °C. The initial temperature of the GC oven was set at 40 °C and held for 2 min before increasing to 220 °C at a rate of 5 °C/min and held for 4 min with a total run time of 42 min. A solvent delay was set for the first 4 min and the MS was operated in electron impact ionization EI+ mode, scanning from ion mass fragments 10–300 m/z with an inter scan delay of 0.1 s and a resolution of 1000 at FWHM (Full Width at Half Maximum). The helium gas flow rate was set at 1 ml/min. Urine samples were randomly analysed by headspace-SPME-GC-MS within 14 h following their treatment.

### Experimental conditions

The following treatments or sample preparation methods were applied to the urine samples collected from each volunteer prior to analysis by headspace-SPME-GC-MS: Fresh, where five aliquots were kept at room temperature and quickly analysed after collection; Frozen, where five aliquots were frozen at −80 °C and defrosted; Freeze-dry, where five aliquots were frozen at −80 °C and freeze-dried for 18 h; NaCl, where five aliquots were frozen at −80 °C, defrosted and treated with 1 ml of salt solution (i.e. saturated NaCl); HCl, where five aliquots were frozen at −80 °C, defrosted and treated with 1 ml of acid solution (i.e. HCl 5M); and NaOH, where five aliquots were frozen at −80 °C, defrosted and treated with 1 ml of base solution (i.e. NaOH 5M). In total, 15 samples of each treatment, five samples from each volunteer, were analysed by headspace-SMPE-GC-MS. The dry residue of each freeze-dried urine was directly analysed by headspace-SMPE-GC-MS with no addition of water or any other substance. We compared the number of VOCs identified, the classes of compounds identified and the variability in metabolite quantification when using each treatment. In addition, we compared the GC column degradation promoted by each treatment.

### Reference solution

Although the urine samples were randomly analysed, we assessed the stability of the headspace-SPME-GC-MS analysis method throughout the study by preparing a stock reference solution containing four compounds dissolved in water: 2-pentanone (CAS 107-87-9), pyridine (CAS 110-86-1), benzaldehyde (CAS 100-52-7) and indole (CAS 120-72-9). A 2 ml aliquot of this reference solution was then analysed on the days the urine samples were analysed. These compounds were selected as reference compounds as their retention times were considerably spread across the GC-MS run. A single stock of reference solution was prepared and used throughout the experiment. The stock solution was stored at room temperature.

### Laboratory air

In order to correct the results for potential air contaminants, samples of the laboratory air were analysed among urine samples. A total of 22 laboratory air samples were analysed throughout the study. Compounds found in more than 50 % of the air samples (Additional file 1) were considered contaminants and were removed from the data analysis.

### Mass spectral library

Two mass spectral libraries were built for this study (Additional files 2 and 3), one for processing samples from the reference solution and another for processing urine samples. They were both built using the Automated Mass Spectral Deconvolution System (AMDIS-version 2.71, 2012) in conjunction with the NIST mass spectral library (version 2.0, 2011). The AMDIS configuration used is available through Additional file 4.

### Data analysis

The GC-MS data were processed using AMDIS in conjunction with the R package Metab [22]. All statistics were performed using R software [23]. A total of 90 urine samples were analysed by headspace-SPME-GC-MS, 30 samples per volunteer (Additional file 5). Outlier samples were those found to contain considerably fewer metabolites in comparison to the rest of the technical replicates. Principal component analysis was used to support the identification of outliers. These were removed from the analysis and comprised of seven samples from volunteer one (one frozen sample, two samples with NaCl, two samples with HCl and two freeze-dried samples) and two samples from volunteer three (one fresh sample and one sample with HCl). Every treatment tested is represented by a minimum of three urine samples. The *p* values lower than 0.05 were considered as significant.

#### Metabolite identification

Initially, the number of compounds identified per sample was determined for each volunteer. For an overall comparison across treatments, the number of compounds identified for a volunteer for all the different treatments tested was divided by the average number of compounds identified for this specific volunteer when using fresh urine. Compounds present in less than 30 % of the samples within a particular condition tested were considered false positives and, thus, were removed from the analysis. In this case, the levels detected for this specific compound were replaced by NA within samples of this particular condition. A Shapiro test indicated that the number of compounds identified per condition was not normally distributed. Thus, the Mann–Whitney U test was applied for comparing the number of compounds identified across treatments. The identified compounds were divided in chemical classes according to their functional groups. A single compound may have multiple functional groups, thus, it may be part of multiple chemical classes.

#### Metabolite quantification

The coefficient of variation (CV) (i.e. standard deviation of abundance divided by the mean abundance and multiplied by 100) was calculated per volunteer and per treatment for each compound identified. The CVs associated with each volunteer were then combined per treatment and the proportion of compounds showing CVs lower than 30 % was calculated. In addition, the proportion of compounds showing a CV of less than 30 % across treatments was compared statistically using 2-sample test for equality of proportions with continuity correction through the R function prop.test.

#### Polysiloxane or column degradation

Polysiloxanes, as part of the silicone septa, part of the SPME fibre or stationary phase of the GC column can degrade, resulting in its de-polymerisation and production of volatile siloxanes. In order to identify if the different sample treatments tested may promote degradation, we compared the abundances of compounds originating from column degradation across treatments. Siloxanes containing the ion fragment 73 were defined as compounds originating from decomposition [24]. Student t test and one-way analysis of variance (ANOVA) were applied on the log transformed abundances of compounds in order to assess statistical differences between treatments. For this analysis, samples that received the same treatment were considered as belonging to the same data class or experimental condition, disregarding the volunteer id. Compounds present in less than 30 % of the samples of all the treatments tested were considered as false positives and, thus, were removed from the analysis (Additional file 6).

## Conclusion

For most metabolomics studies, the ideal sample preparation method would be easy to perform, quick, cheap, accurate, able to recover a high number of compounds of multiple classes and be highly reproducible. The results presented here indicate that HCl and Freeze-dry were the methods that reported the most enhanced profiles of VOCs, with Freeze-dry covering a slightly higher number of compound classes, showing better repeatability across replicates and producing a significantly lower level of most polysiloxane degradation products. In addition, Freeze-dry is considerably easier to perform, safer and can be cheaper than alkaline or acidic treatments if a freeze-drier is available, which is generally the case for most universities with laboratories of chemistry and/or biology. The use of multiple sample preparation methods is certainly recommended for untargeted metabolomics when financial resources are available and the study design allows it. The results reported here indicate that freeze-drying urine samples for SPME-GC-MS analysis is a potential alternative sample preparation method. A larger experiment using internal standard will allow to confirm the results reported here and further understand chemistry and physics behind free-drying urine samples for SPME-GC-MS analysis.

## Additional files

10.1186/s13065-016-0155-2 Air contaminants. This csv file contains the metabolites found as air contaminants. These metabolites were detected in laboratory air samples analysed by SPME-GC-MS. This file can be visualised using a text editor.
10.1186/s13065-016-0155-2 Mass spectral library for processing reference solution. This msl file is the AMDIS library containing the compounds present in the reference solution used in this study. This file can be visualised using a text editor or it can be loaded into AMDIS software. Please see the user manual provided with AMDIS to obtain all the necessary information to load a new library.
10.1186/s13065-016-0155-2 Mass spectral library for processing urine samples. This msl file is the AMDIS library built to identify compounds in the urine samples analysed in this study. This library was built using NIST mass spectral library version 2.0, 2011. This file can be visualised using a text editor or it can be loaded into AMDIS software. Please see the user manual provided with AMDIS to obtain all the necessary information to load a new library.
10.1186/s13065-016-0155-2 AMDIS configuration. This ini file contains the settings of the AMDIS software used in this study. Please see the user manual provided with AMDIS to obtain all the necessary information to use this configuration file when analysing GC-MS samples with AMDIS.
10.1186/s13065-016-0155-2 Metabolite found in urine samples. This csv file contains the metabolites found in the samples analysed in this study. This file can be visualised using a text editor.
10.1186/s13065-016-0155-2 Column degradation. This csv file contains the metabolites defined as product of GC column degradation. This file can be visualised using a text editor.

## Abbreviations

VOCs: volatile organic compounds
GC-MS: gas chromatography - mass spectrometry
SPME: solid phase micro extraction
NaCl: sodium chloride
NaOH: sodium hydroxide
HCl: hydrochloric acid
